# Gross Dissection of the Stomach of the Lobster, *Homarus Americanus*

**DOI:** 10.3791/1320

**Published:** 2009-05-22

**Authors:** Hilary S. Bierman, Anne-Elise Tobin

**Affiliations:** Volen Center for Complex Systems, Brandeis University

## Abstract

The stomach of the American lobster (*Homarus americanus*) is located in the cephalothorax, between the rostrum and the cervical groove. The anterior end of the stomach is defined by the mouth opening and the posterior end by the bottom of the pylorus.  Along the dorsal side of the stomach lies the stomatogastric nervous system (STNS). This nervous system, which contains rhythmic networks that underlie feeding behavior, is an established model system for studying rhythm generating networks and neuromodulation ^1,2^.  While it is possible to study this system *in vivo*^ 3^, the STNS continues to produce its rhythmic activity when isolated *in vitro*. In order to study this system *in vitro* the stomach must be removed from the animal. This video article describes how the stomach can be dissected from the American lobster. In an accompanying video article^4^ we demonstrate how the STNS can be isolated from the stomach.

**Figure:**
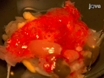


## Protocol

Before dissection the lobster should be buried in ice for 30 minutes to anesthetize the animal.
The sex of the lobster can be determined by looking at the shape of the first swimmeret. Males have boney swimmerets and females have feathery swimmerets.
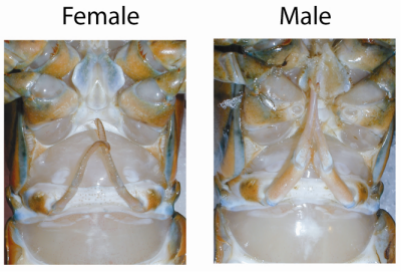
Isolate the cephalothorax.
  Use a twisting motion to rip off the front claws.
  Remove the abdomen (tail portion) by using the large scissors to make a deep cut between the cephalothorax and abdomen. This cuts the giant fiber tracts controlling the tail flip response. Then proceed to cut off the abdomen.
  Remove the antennae by trimming them off with scissors, and twist off the legs and the third maxillipeds.
Remove the carapace from around the stomach, (i.e. the head region between the cervical groove and the rostrum).
  Starting on the lateral edge of this section use rongeurs and spatula to carefully remove the hard carapace, while leaving the reddish underlying hypodermis intact. The spatula is useful for separating the layers so that the carapace can be broken off with the rongeurs.
  Moving toward the eyes you find bilateral lightly calcified small oval plates (IIIa ossicles).  As close as possible to these ossicles, carefully detach the muscle from the ossicles using the small scissors.
  Using spatula and rongeurs remove carapace from between the eyes and the caudal portion (approx. 1 centimeter) of the rostrum.
Detach the face (region including eyes, and rostrum).
  Using small scissors make a horizontal cut across the most rostral exposed tissue in the rostrum to detach it from the exoskeleton. Then use edge of scissors and/or spatula to carefully roll tissue caudally, just past the level of the IIIa ossicles.
  Starting dorsally, use small scissors to detach remaining tissue from the face exoskeleton. Cut as close to the hard carapace as possible, stopping when you get near the center and attachment of the mouth.
  From ventral view the mandibles should be easily visible. Detach remaining maxillipeds.
  Locate soft lip under the mandibles.
  Make lateral cuts to detach the face while keeping the lip attached to the stomach. (There is a slightly curved bluish outer mouth part that can be used as a guide.)
Detach and remove the stomach.
  Hold the lobster in the palm of your hand, ventral side up and so that the anterior end is toward your elbow.
  Attach a hemostat to the lip and let gravity pull the hemostat and lip down and away from the ventral body wall.
  Using small scissors cut out mandibles.
  Locate the dorsal side of the ventral body wall, which forms a hard “shelf” ventral to lip and stomach. Cut just below the "shelf", very close to the body wall, across the entire medial-lateral midline. The entire ventral side of the stomach should now be detached from the body wall.
  Turn the lobster so that the dorsal side is up. Cut along the rostral edge of the cervical groove, where the carapace was removed, and gently pulls the stomach out.
  Detach any extra intestines that remain attached to the stomach.
Open the stomach, and prepare it for pinning.
  Hold the stomach in the palm of your hand with the hypodermis-side down.  The hemostat should still be attached to the lip and should lie between your fingers.
  Use small scissors to make a shallow anterior-to-posterior cut from the mouth opening (below the lip) down through the ventral side of the pylorus.
  To further open the stomach, make two lateral cuts along the ossicles running across the center of the stomach.
  Cold saline may be poured into the stomach to improve visibility and expel stomach contents.
  In order to allow the preparation to lay flat in the dissecting dish use the small scissors to trim the three gastric teeth.
Pin the stomach down in the dissection dish.
  Place the stomach, hypodermis-side up, in a saline-filled dissection dish.
  Place insect pins at the lip and the four corners created when the stomach was opened up.  For the anterior corners place pins high and close to the edge, as not to disturb commissural ganglia or inferior oesophageal nerves. For the posterior corners place pins slightly high as not to disturb the pyloric dilator muscle.

## Discussion

In our laboratory dissection of the lobster stomach is done to access the stomatogastric nervous system, a network that controls the rhythmic contractions of the stomach.  The anatomy of lobster stomach has been well described previously^5-7^, and we recommend becoming familiar with its anatomy.  Doing so will reduce the risk of damaging the delicate nerves and muscle.

Upon completion of the stomach dissection, various portions of the STNS may be further dissected.  For example to study neuromuscular junctions, one of the STNS nerves and the muscle it innervates may be isolated.  The somata of neurons in the stomatogastric ganglion may be accessed for recordings or molecular work by isolating the stomatogastric ganglion.  The dissection of the STNS, including the stomatogastric ganglion and nerves, has been documented in a companion JoVE article^4^.
